# Sub-Cluster Identification through Semi-Supervised Optimization of Rare-Cell Silhouettes (SCISSORS) in single-cell RNA-sequencing

**DOI:** 10.1093/bioinformatics/btad449

**Published:** 2023-07-27

**Authors:** Jack R Leary, Yi Xu, Ashley B Morrison, Chong Jin, Emily C Shen, Peyton C Kuhlers, Ye Su, Naim U Rashid, Jen Jen Yeh, Xianlu Laura Peng

**Affiliations:** Lineberger Comprehensive Cancer Center, University of North Carolina at Chapel Hill, Chapel Hill, NC 27599, United States; Department of Biostatistics, University of Florida, Gainesville, FL 32603, United States; Department of Pharmacology, University of North Carolina at Chapel Hill, Chapel Hill, NC 27599, United States; Lineberger Comprehensive Cancer Center, University of North Carolina at Chapel Hill, Chapel Hill, NC 27599, United States; Department of Biostatistics, University of North Carolina at Chapel Hill, Chapel Hill, NC 27599, United States; Lineberger Comprehensive Cancer Center, University of North Carolina at Chapel Hill, Chapel Hill, NC 27599, United States; Lineberger Comprehensive Cancer Center, University of North Carolina at Chapel Hill, Chapel Hill, NC 27599, United States; Department of Biostatistics, University of North Carolina at Chapel Hill, Chapel Hill, NC 27599, United States; Lineberger Comprehensive Cancer Center, University of North Carolina at Chapel Hill, Chapel Hill, NC 27599, United States; Lineberger Comprehensive Cancer Center, University of North Carolina at Chapel Hill, Chapel Hill, NC 27599, United States; Department of Biostatistics, University of North Carolina at Chapel Hill, Chapel Hill, NC 27599, United States; Lineberger Comprehensive Cancer Center, University of North Carolina at Chapel Hill, Chapel Hill, NC 27599, United States; Department of Pharmacology, University of North Carolina at Chapel Hill, Chapel Hill, NC 27599, United States; Department of Surgery, University of North Carolina at Chapel Hill, Chapel Hill, NC 27599, United States; Lineberger Comprehensive Cancer Center, University of North Carolina at Chapel Hill, Chapel Hill, NC 27599, United States; Department of Pharmacology, University of North Carolina at Chapel Hill, Chapel Hill, NC 27599, United States

## Abstract

**Motivation:**

Single-cell RNA-sequencing (scRNA-seq) has enabled the molecular profiling of thousands to millions of cells simultaneously in biologically heterogenous samples. Currently, the common practice in scRNA-seq is to determine cell type labels through unsupervised clustering and the examination of cluster-specific genes. However, even small differences in analysis and parameter choosing can greatly alter clustering results and thus impose great influence on which cell types are identified. Existing methods largely focus on determining the optimal number of robust clusters, which can be problematic for identifying cells of extremely low abundance due to their subtle contributions toward overall patterns of gene expression.

**Results:**

Here, we present a carefully designed framework, SCISSORS, which accurately profiles subclusters within broad cluster(s) for the identification of rare cell types in scRNA-seq data. SCISSORS employs silhouette scoring for the estimation of heterogeneity of clusters and reveals rare cells in heterogenous clusters by a multi-step semi-supervised reclustering process. Additionally, SCISSORS provides a method for the identification of marker genes of high specificity to the cell type. SCISSORS is wrapped around the popular Seurat R package and can be easily integrated into existing Seurat pipelines.

**Availability and implementation:**

SCISSORS, including source code and vignettes, are freely available at https://github.com/jr-leary7/SCISSORS.

## 1 Introduction

Single-cell RNA-sequencing (scRNA-seq), which has garnered tremendous interest in the field of biomedical research in the past decade, enables the study of the transcriptome of a tissue or organ at an unprecedented resolution (Tang *et al.* 2009, Zheng *et al.* 2017). Compared with bulk RNA-seq, which can only capture the average of gene expressions in a sample, scRNA-seq enables the profiling of the transcriptome in each individual cell simultaneously, which can theoretically lead to a more comprehensive and exhaustive understanding of each cell type. However, the computational analysis of scRNA-seq data is often challenging due to its sparse, noisy, and high dimensional features (Bacher and Kendziorski 2016, Lähnemann *et al.* 2020).

As identifying cell populations is one of the principal goals of performing scRNA-seq, accurate analytical approaches for clustering cells are critical. To this end, a wide variety of clustering algorithms are employed in scRNA-seq data analysis. For example, *k*-means, a popular algorithm used widely across many fields, has been implemented as part of the methods, such as GiniClust3, SC3, and RaceID (Grün *et al.* 2015, Kiselev *et al.* 2017, Dong and Yuan 2020). However, one of the assumptions of the *k*-means clustering method is that features have spherical covariance (Bock 1996). This is easily violated by the sparse scRNA-seq data, which feature non-independent, co-expressing genes that exhibit different variances depending on which cell types they are expressed in. In addition, *k*-means tends to produce similarly sized clusters due to the objective function it minimizes, the sum of squared errors; this is often sub-optimal for biological data where cell types appear with widely varying frequencies depending on the species, organ, and phenotype being studied (Pollard 1981, Bock 1996, Andrews and Hemberg 2018). This may negatively impact the performance of *k*-means and is exacerbated in the presence of rare cell types. Another method, Spectrum, has been created to accommodate negative-binomially distributed single-cell counts through its implementation of spectral clustering on only 300–8500 cells and 100 highly variable genes (John *et al.* 2020). However, spectral clustering is computationally expensive, with runtime scaling cubically with sample size, which is a growing constraint as single-cell datasets have grown from thousands to millions of cells (Lähnemann *et al.* 2020). Recently, the graph-based Louvain modularity optimization clustering algorithm as implemented in Seurat and Scanpy has been shown to be very accurate and achieves reasonable runtimes (Blondel *et al.* 2008, Duo *et al.* 2018, Wolf *et al.* 2018, Stuart *et al.* 2019, Hao *et al.* 2021). The IKAP method is built around Seurat and begins by first overclustering and then transitioning to a coarser clustering by iteratively combining the clusters whose centers are closest (Chen *et al.* 2019). However, IKAP performance may be limited on highly heterogeneous samples, such as tumors, or developmental datasets where expression is expected to exist on a gradient (Chen *et al.* 2019). Similar to IKAP, the recently developed MultiK method attempts to find the true number of clusters *K* in a dataset by iteratively testing many combinations of the resolution parameter *r* (Liu *et al.* 2021). Both IKAP and MultiK are time-intensive due to the design of iterating over parameters (Chen *et al.* 2019, Liu *et al.* 2021). In addition, SAFE-clustering is an ensemble method that derives a consensus clustering from the results of several different clustering methods (Yang *et al.* 2019).

Besides the general need for calling cell types in a dataset, the identification of rare cell types may be of particular interest. A handful of clustering methods have been developed specifically for this purpose, as the identification of rare cell types from scRNA-seq data is challenging and necessitates special methodologies. For example, GiniClust3 identifies marker genes through Gini indexing, then uses a graph-based clustering algorithm in an attempt to find rare cell types (Dong and Yuan 2020). However, it uses *k*-means as part of the final consensus clustering, which may compromise the accuracy of its results as the *k*-means assumptions are often violated as previously described. This is the case for CellSIUS and RaceID as well, which were designed for rare cells but also employ the *k*-means algorithm for clustering (Grün *et al.* 2015, Wegmann *et al.* 2019).

The gap in methods for rare cell identification motivated our development of SCISSORS, which is wrapped around Seurat and strives to achieve a biologically optimal clustering. SCISSORS employs carefully designed initial clustering and reclustering steps, which ensures the identification of cell types that are of extremely low abundance. In SCISSORS, the silhouette coefficient is used to allow the systematic estimation of intra-cluster heterogeneity, which can help determine if reclustering is needed for a cluster when biological information is insufficient. In the reclustering step, SCISSORS efficiently tests several default or user-defined combinations of parameters and determines the best parameter set, which systematically optimize the final clusters. By applying SCISSORS to a peripheral blood mononuclear cell (PBMC) dataset, as well as a pancreatic ductal adenocarcinoma (PDAC) dataset, we demonstrated that SCISSORS is able to identify cells that were overlooked in other analyses possibly due to their rare representation in the dataset. In this study, the “identification of rare cells” refers to the more rigorous statement of “the identification of evidence for the existence of rare cells” if not specified otherwise.

## 2 Materials and methods

### 2.1 Silhouette score

SCISSORS uses silhouette scores (Rousseeuw 1987) based on the cosine distance between cells to estimate the heterogeneity of derived clusters and to evaluate parameter sets during reclustering. To measure the dissimilarity or distance between two non-zero vectors, the cosine distance is defined as: where *a* and *b* are two vectors each of length *n*. The mean distance from cell *i* to all other cells *j* in its cluster *C_m_* is defined as: where *d(i, j)* is the cosine distance from cell *i* to cell *j*. The minimum mean distance from cell *i* to all points in all other clusters is then calculated as:


(1) da, b=1-∑i=1naibi ∑i=1nai2 × ∑i=1nbi2,

(2) ai=1Cm-1∑j∈Ck,i≠jdi,j,

(3) bi=minl≠m⁡1Cl∑j∈Cldi, j.

The silhouette score of each individual cell in cluster *C_m_* is then computed using the following:


(4) silhouettei=ai-bimaxai,bi.

The silhouette coefficient of cluster *C_m_* is given by: where *N* is the number of cells in cluster *C_m_*.


(5) silhouetteCm=N-1∑i=1Nbi-aimax⁡ai, bi,

### 2.2 Marker gene identification

Marker genes for broad clusters or subclusters were identified using a specifically designed integrative method, which is included as a function in SCISSORS. In this method, a given cluster or subcluster is first compared to background clusters, which may be the rest of the clusters or other closely related subclusters, to generate a candidate gene list. Subsequently, this candidate gene list is filtered by removing highly expressed genes in the clusters that are not of interest. The highly expressed genes are defined as the top expressed (top 10% by default) by averaging all the cells within each cluster; a user-defined percentile cutoff can also be supplied. For each of the clusters and subclusters in the PBMC3K and PDAC datasets, marker genes were identified using this function in SCISSORS, the details of which are included in Supplementary Table S1.

### 2.3 Package access

SCISSORS, including source code and analysis vignettes for simulation and biological datasets, is freely available at https://github.com/jr-leary7/SCISSORS.

## 3 Results

### 3.1 The SCISSORS framework

**Figure 1.**
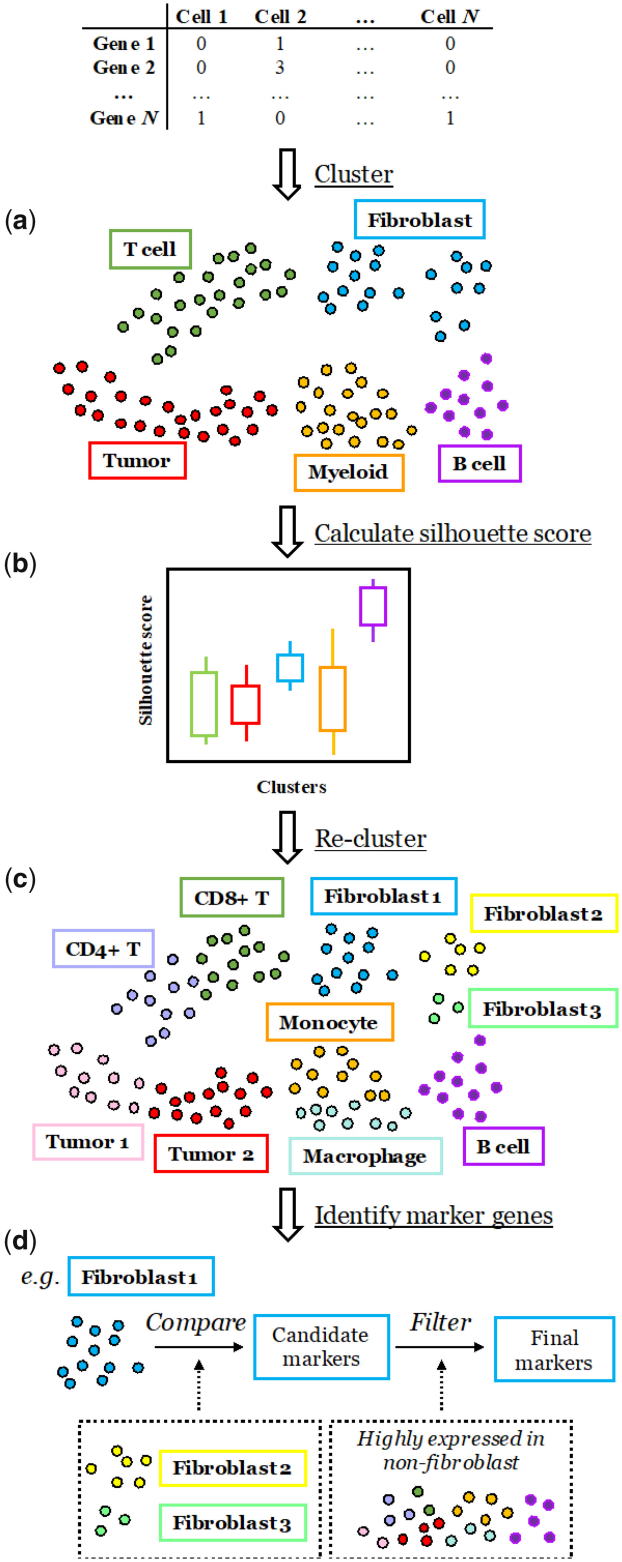
Overview of the SCISSORS framework. SCISSORS is a wrapper of the Seurat package and integrates multi-step semi-supervised clustering to identify cells of low abundance (rare cells) in scRNA-seq datasets. (a) SCISSORS first coarsely defines broad clusters using Louvain clustering in Seurat with conservative parameters. (b) Then, silhouette scores are calculated to infer the heterogeneity of each broad cluster. Lower silhouette score indicates higher heterogeneity of a cluster. The median silhouette score combined with biological knowledge aids the user in determining reclustering candidates. (c) In the reclustering step, combinations of reclustering parameters are tested, and the set that maximizes the silhouette scoring metric is chosen as optimal. (d) Finally, marker genes are identified for each of the clusters and subclusters to facilitate cell type annotation and further study. For the accurate identification of marker genes in subclusters or rare cells, SCISSORS integrates a carefully designed function, where the subcluster is first compared to the closely related clusters followed by a filtering step to remove highly expressed genes in the rest of the clusters

The motivation of SCISSORS is rooted in the fact that cell types in a given dataset are of variable abundance, and the divergence levels between broad cell types versus between subpopulations within the same broad cell type are not comparable. This has made the estimation of the true number of cell clusters *K* difficult, especially when cell types of low abundance exist in the dataset. To solve this problem, SCISSORS proposes to execute a multi-step process that splits potentially heterogenous cell clusters into subclusters, which leads to the identification of cell types of low abundance (Fig. 1).

With a preprocessed count matrix, the SCISSORS framework first performs an initial clustering step to define broad clusters using conservative parameters (Fig. 1a). To evaluate the level of heterogeneity of each broad cluster, SCISSORS calculates the silhouette score of each cell, which measures how well cells fit within their assigned clusters (Rousseeuw 1987) (Fig. 1b). A lower median silhouette score or large variations in the score distribution of a cluster may indicate higher heterogeneity in that cluster. This measurement aids the determination of heterogeneous broad clusters in a systematic and reproducible way, especially when biological information is lacking. The selection of heterogeneous broad clusters as reclustering candidates avoids overclustering on originally homogeneous or biologically unimportant cell clusters.

In the second round of clustering, SCISSORS re-identifies HVGs from all the gene features (Hafemeister and Satija 2019), considering only the selected candidate broad cluster(s) instead of using the genes identified in the initial processing of the entire dataset. By doing so, SCISSORS avoids losing features that are essential in distinguishing closely related cell subpopulations from one another but are minimally representative on the scale of the entire dataset. SCISSORS then enumerates several combinations of clustering parameters to achieve optimal performance by computing and comparing their silhouette coefficients. The identified resultant subclusters can then be integrated back into the original dataset by SCISSORS and visualized (Fig. 1c). Additional reclustering can also be performed on subclusters to further subdivide cell subpopulations until the cell types are considered fully explored.

SCISSORS also integrates a function to aid with the identification of marker genes for rare cell types (Fig. 1d). This function first compares the rare cells to other cells that fall into the same broad cluster(s) or are empirically determined as closely related cell subpopulations to derive a candidate gene list. This is because comparison within the mixture of the entire dataset may miss small differences between rare cells and their closely related cells. Then, this function filters the candidate gene list by removing the highly expressed genes in all the other clusters in the dataset. By doing so, SCISSORS can accurately identify distinguishing and highly cell-subpopulation-specific marker genes for rare cell types.

### 3.2 Evaluation of SCISSORS in simulation test

**Figure 2.**
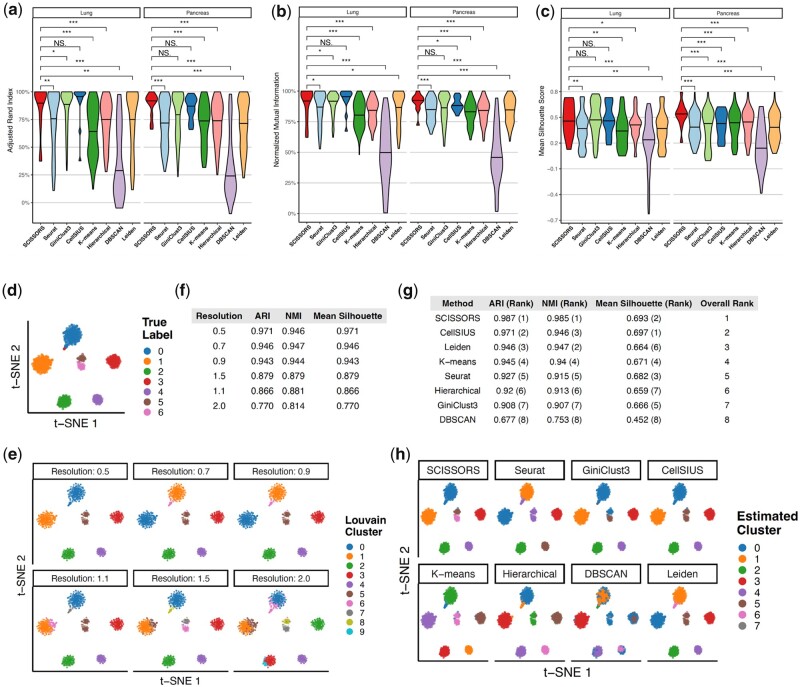
Evaluation of SCISSORS in simulation test. (a–c) Distributions of ARI, NMI, and silhouette score for runs of each clustering method across all simulated datasets combined. (d) *t*-SNE embedding of one representative simulated dataset with seven clusters as ground truth. Note that Clusters 5 and 6, which are located closely together in the dimension-reduced space, are composed of fewer cells than all other clusters, mimicking clusters of rare cells. (e and f) Clustering result and evaluation metrics for naively increasing the resolution parameter (*r*) in the Seurat method in the representative simulated dataset. (g and h) Evaluation metrics and clustering results for each method in the representative simulated dataset

To evaluate the performance of SCISSORS systematically, we generated a collection of simulation datasets based on different combinations of parameters using two references datasets respectively, namely a human lung cancer dataset (Zilionis *et al.* 2019) and a human normal pancreas dataset (Baron *et al.* 2016). We analyzed and clustered these simulation datasets, where ground truth labels are available, by different methods including SCISSORS, Seurat (Louvain clustering), GiniClust3, CellSIUS, three representative basic algorithms (*k*-means, hierarchical, and DBSCAN), and Leiden clustering, which is generally comparable to Louvain clustering (Ward 1963, Hartigan and Wong 1979, Ester *et al.* 1996, Blondel *et al.* 2008, Traag *et al.* 2019, Wegmann *et al.* 2019, Dong and Yuan 2020, Hao *et al.* 2021). To derive a distribution of performance instead of a single point estimate for an unbiased comparison, iterations of common and reasonable parameters were used for each method (Supplementary Table S2). Comparing against the ground truth, SCISSORS showed the best recapitulation of the clustering labels measured by ARI and NMI in datasets simulated based on both the human lung cancer dataset and the human normal pancreas dataset (Fig. 2a and b). In addition, SCISSORS had the highest median silhouette score (Fig. 2c), indicating that resultant clusters from SCISSORS had the best cluster separations or lowest heterogeneity, indicating a lower possibility of the existence of cell subpopulations. Of note, although SCISSORS is compatible with being supervised, the simulation analysis using SCISSORS utilized the completely unsupervised mode, without setting any parameters *a priori*.

To further illustrate the superiority of SCISSORS in rare cell identification, we zoomed in on its clustering performance in one of the simulated datasets from the pancreas reference. This simulation is composed of 996 cells and 15 270 genes overall; it contains seven distinct true clusters, with two very small clusters placed closely together in the low-dimensional space, mimicking the situation of rare cell identification (Fig. 2d). Simply increasing resolution *r* from 0.5 to 2.0 led first to a larger broad cluster being broken up. Then at around resolution *r *=* *1.5, where two clusters of “rare” clusters were separated, another broad cluster (on the left) was wrongly split (Fig. 2e). In addition, the clustering with the lowest resolution tested (0.5) had the highest ARI, NMI, and silhouette score, indicating that simply increasing the resolution does not lead to a better estimated clustering (Fig. 2f). Then, we compared the performance of the eight above-mentioned methods on clustering this specific dataset. Out of all eight methods, SCISSORS achieved the highest ARI (0.987), highest NMI (0.985), and second highest silhouette score (0.693) (Fig. 2g). In addition, SCISSORS was the only method that successfully separated the two rare clusters (Fig. 2h).

### 3.3 Application of SCISSORS to the PBMC3K dataset

**Figure 3.**
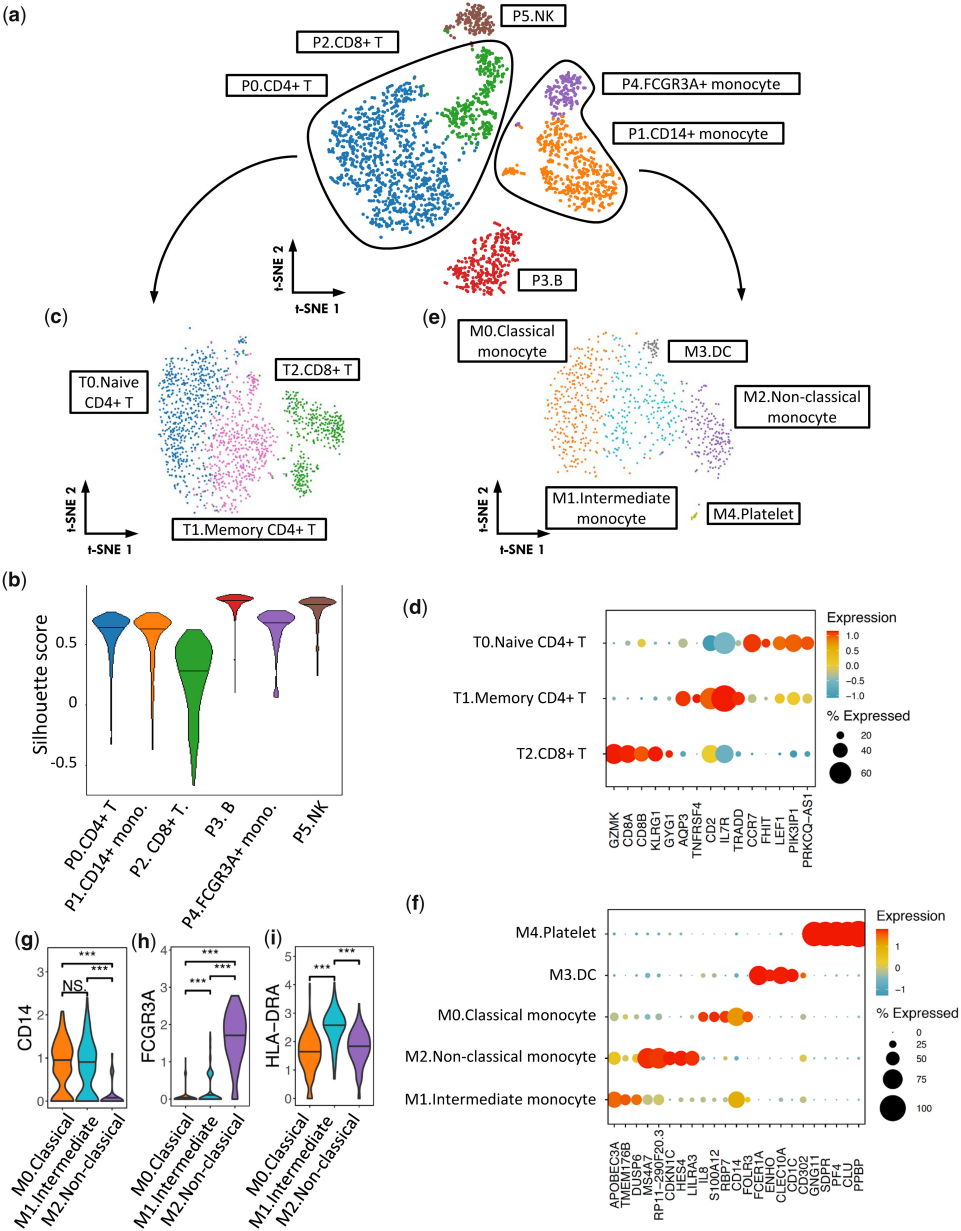
Application of SCISSORS to the PBMC3K dataset. (a) Initial conservative clustering on the PBMC3K dataset. Six broad clusters were identified. (b) Violin plot for silhouette scores in each broad clusters. (c) Reclustering of broad clusters containing CD4+ and CD8+ T cells. (d) Top 5 marker genes identified by SCISSORS, ranked by fold-change. (e) Reclustering of candidate broad clusters containing CD14+ and FCGR3A+ monocytes. (f) Top 5 marker genes identified by SCISSORS, ranked by mean log2 fold-change. (g–i) Marker gene expression of monocyte subtypes in the three monocyte subclusters. ***P < .05, Wilcoxon rank-sum test

We applied SCISSORS to the widely used PBMC3K dataset, which was previously analyzed in a tutorial for Seurat (https://satijalab.org/seurat/articles/pbmc3k_tutorial.html). We used SCISSORS to perform an initial clustering, which yielded six clusters (Fig. 3a) with varying silhouette scores (Fig. 3b) and marker genes (Supplementary Fig. S1). Clusters P3 and P5 were annotated as being composed of B cells and natural killer cells, respectively (Fig. 3a and Supplementary Fig. S1), and exhibited high silhouette scores, suggesting that they were relatively homogeneous (Fig. 3b). Clusters P0 and P2 were annotated as CD4+ and CD8+ T cells separately (Fig. 3a and Supplementary Fig. S1), which were more heterogeneous as defined by lower silhouette scores (Fig. 3b). This, along with their biological similarity, led us to combine Clusters P0 and P2 for reclustering using SCISSORS. SCISSORS reclustering identified three subclusters, namely Naive CD4+ T cells, Memory CD4+ T cells, and CD8+ T cells (Fig. 3c and d), which reproduced the Seurat results.

**Figure 4.**
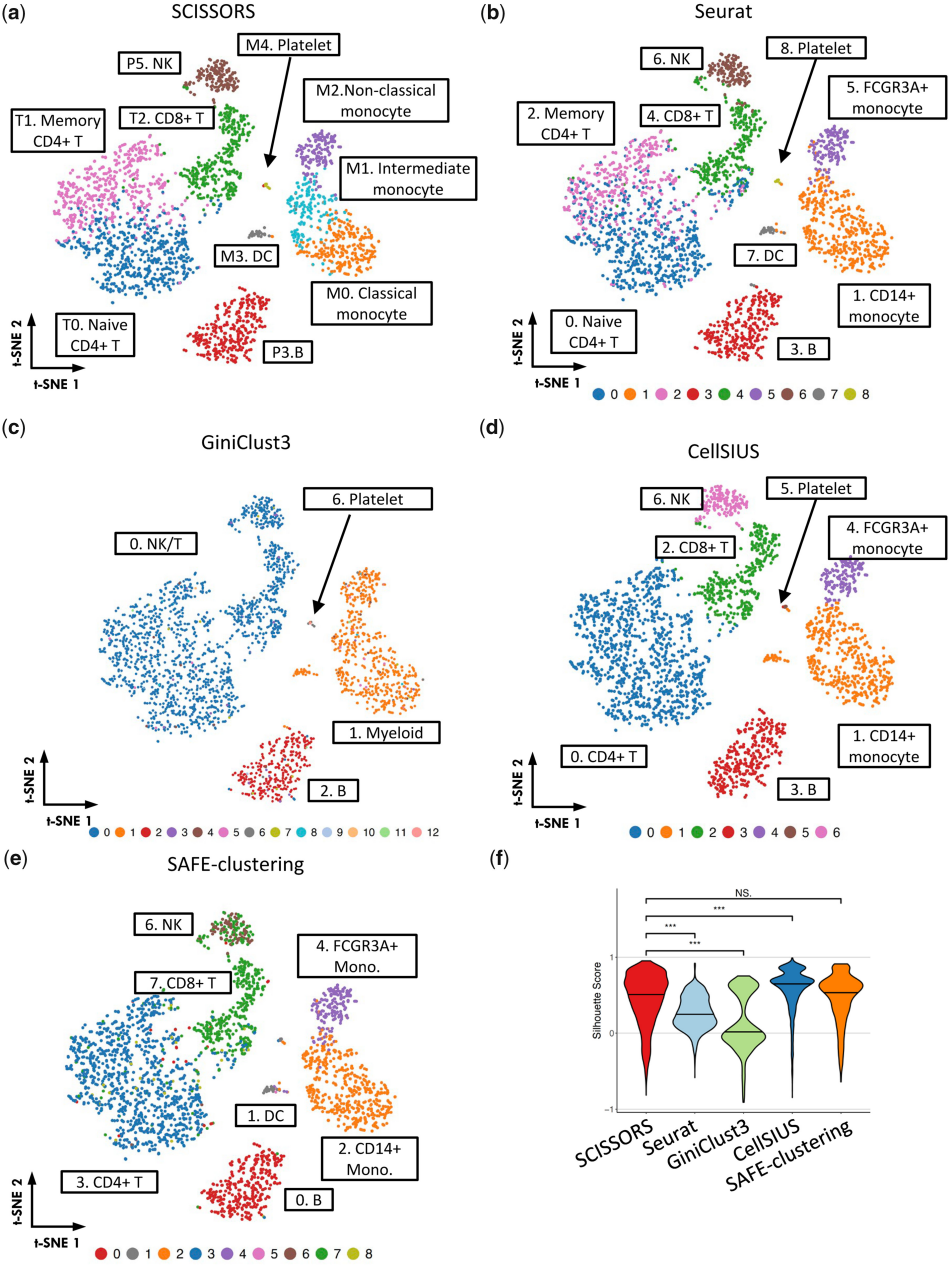
Evaluation of SCISSORS in the PBMC3K dataset. (a) Final clusters, with broad clusters without reclustering and subclusters derived by reclustering in SCISSORS. (b–e) Clusters identified by using comparable methods, including Seurat, GiniClust3, CellSIUS, and SAFE-clustering, respectively. *t*-SNE coordinates were derived in SCISSORS analysis in (a). (f) Violin plots of silhouette score distributions by each method tested. ***P < .05, Wilcoxon rank-sum test

Clusters P1 and P4 were both annotated as containing myeloid cells and were subjected to reclustering (Fig. 3a and Supplementary Fig. S1). As a result, five subclusters were identified (Fig. 3e). Among them, SCISSORS reproducibly defined dendritic cells (DC) and platelets as Seurat (Fig. 3e and f). The remaining cells were monocytes, which were reclustered into three subclusters (M0, M1, and M2) by SCISSORS (Fig. 3e and f). Human monocytes are known to be divided into three major populations, namely classical, non-classical, and intermediate monocyte, which are primarily distinguished by different levels of CD14 and CD16 (FCGR3A) (Kapellos *et al.* 2019, Ong *et al.* 2019). Among the three monocyte subclusters identified by SCISSORS, subcluster M2 was annotated as non-classical monocyte, as it showed the highest level of FCGR3A (CD16) and a decreased level of CD14 (Kapellos *et al.* 2019, Ong *et al.* 2019) (Fig. 3g and h). Interestingly, in the previously annotated CD14+ monocytes, SCISSORS identified a subcluster M1, which showed a similarly high level of CD14 (Fig. 3h) with subcluster M0, but an intermediate level of CD16 (FCGR3A) compared with subclusters M0 and M2 (Fig. 3h), indicating that this may be an intermediate monocyte cluster. Additionally, M1 showed a significantly higher level of HLA-DR than the rest of the monocyte subclusters (Fig. 3i), indicating that it may consist of intermediate monocytes. Accordingly, we annotated subclusters M0, M1, and M2 as classical monocyte, intermediate monocyte, and non-classical monocyte (Fig. 3e). To our knowledge, the intermediate monocyte cluster was not identified before in this dataset. This demonstrated that SCISSORS can identify cells that may be overlooked by traditional clustering methods due to their low abundance or high degree of similarity to neighboring cell clusters. The annotated subclusters were integrated with the non-reclustered broad clusters, all of which were visualized on the original *t*-SNE embedding (Fig. 4a).

### 3.4 Evaluation of SCISSORS in the PBMC3K dataset

We used the PBMC3K dataset to evaluate the performance of SCISSORS against other comparable methods, namely Seurat, GiniClust3, CellSIUS, and SAFE-clustering (Wegmann *et al.* 2019, Yang *et al.* 2019, Dong and Yuan 2020, Hao *et al.* 2021). Cell clusters were annotated by examining the marker genes and visualized on the same *t*-SNE embedding obtained from SCISSORS application (Fig. 4a–e). As shown above, compared with Seurat, SCISSORS reproducibly found the same eight final clusters, while also identified an extra cluster of intermediate monocytes, M1.Intermediate monocyte (Fig. 4a and b). Clusters identified by GiniClust3 were disparate, with 4 out of 13 clusters matching Seurat labels (Fig. 4c). The remaining 9 clusters with 117 total cells were scattered throughout the *t*-SNE embedding and did not correspond to known cell types (Fig. 4c). CellSIUS clustered the dataset into seven clusters, which reproduced the broad cell types in Seurat labels (Fig. 4d). However, it did not break down them into finer subpopulations, e.g. naive and memory CD4+ T cells were combined as one large CD4+ T cell cluster (Fig. 4d). Compared with SCISSORS specifically, DC, classical monocyte, and intermediate monocyte were merged into the same cluster in CellSIUS (Fig. 4a). With suggested parameters, SAFE-clustering did not separate the CD4+ T cells into their naïve and memory subtypes, and also failed to cluster platelets and DCs accordingly as they were of relatively lower frequency in this dataset (Fig. 4e). Finally, silhouette scores were calculated to evaluate the clustering results from different methods. SCISSORS showed significantly higher median silhouette scores than Seurat and GiniClust3 (Fig. 4f). Although SCISSORS did not show significantly higher silhouette scores than CellSIUS and SAFE-clustering, it better separates cell subpopulations than either method. Altogether, the final SCISSORS-identified clusters were of lower heterogeneity and more meaningful due to its extensive grouping of cells within broad clusters into smaller and biologically relevant subclusters.

### 3.5 Pinpointing overlooked basal-like cells in a PDAC dataset

We applied SCISSORS to a published PDAC dataset (Elyada *et al.* 2019). The initial clustering yielded 13 clusters with varying silhouette scores (Supplementary Fig. S2a and b), indicating varying levels of intra-cluster heterogeneity. Annotation by SingleR (Aran *et al.* 2019) and cell markers (Elyada *et al.* 2019) confirmed the broad clusters of epithelial/ductal cells, activated stellate/mesenchymal stem cells, acinar cells, and multiple types of immune cells (Supplementary Fig. S2c–e). This initial round of clustering and annotation does not aim to exhaustedly determine all possible cell populations. However, the silhouette scores and annotations allowed us to systematically determine candidates for the reclustering analysis.

**Figure 5.**
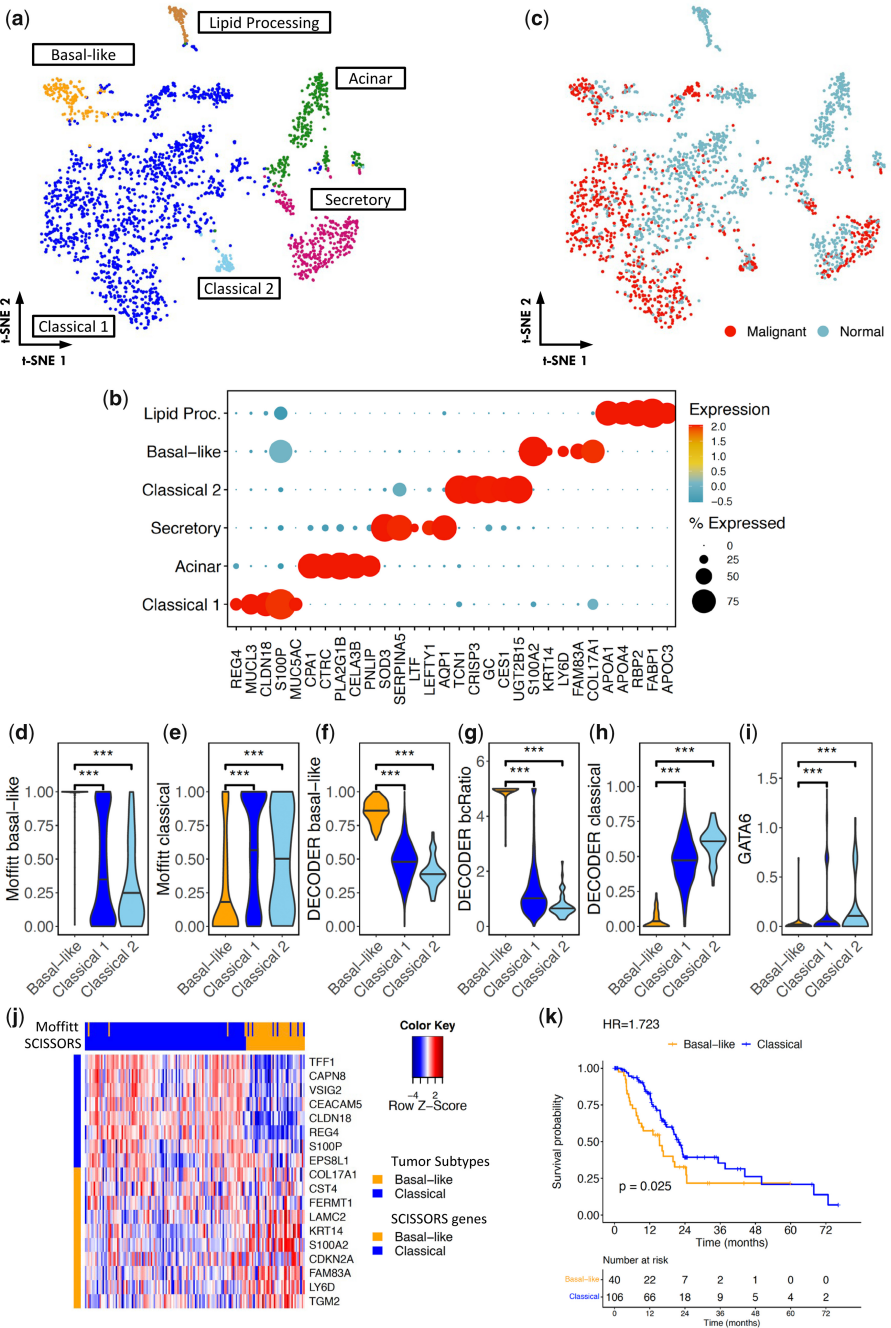
Pinpointing overlooked basal-like cells in a PDAC dataset. (a) Reclustering of the epithelial/ductal cell clusters revealed six subclusters. (b) Top 5 marker genes identified by SCISSORS, ranked by fold-change. (c) CNV analysis for the identification of malignant cells. (d and e) Gene enrichment analysis for each cell in the three tumor subclusters, using VAM scores based on top 25 Moffitt basal-like and top 25 Moffitt classical genes. The identified basal-like cluster showed significantly higher basal-like gene enrichment, but lower classical gene enrichment. (f–h) Tumor subtype analysis in the three tumor subclusters, using DECODER basal-like weights, the ratio between basal-like and classical weights (bcRatio), and classical weights. The identified basal-like cluster showed enriched basal-like weights and bcRatio. (i) GATA6 expression in the identified tumor subclusters. The identified basal-like cluster showed depleted GATA6 expression. (j) Comparison of tumor subtypes called by using SCISSORS derived tumor genes, with published calls derived by the Moffitt schema. Heatmap shows consensus clustering of TCGA PAAD samples using top 10 SCISSORS tumor genes, ranked by fold-change. (k) Kaplan–Meier plots of overall survival in patients with resected PDAC for tumor subtypes derived by using SCISSORS marker genes. Log-rank test was used for survival analysis. ***P < .05, Wilcoxon rank-sum test

Many studies have shown the co-existence of basal-like and classical tumor cells, corresponding to the two intrinsic tumor subtypes found in PDAC patients (Collisson *et al.* 2011, Moffitt *et al.* 2015, Bailey *et al.* 2016, Peng *et al.* 2019, Chan-Seng-Yue *et al.* 2020, Juiz *et al.* 2020, Moncada *et al.* 2020, Rashid *et al.* 2020). To understand the heterogeneity within tumor cells and study their subpopulations, we combined clusters of epithelial cells (Clusters 5 and 9) (Supplementary Fig. S2a) and performed reclustering. This resulted in the identification of the same subclusters as in the original study, as well as an additional subcluster that was not identified before (Fig. 5a and b). Copy number variation (CNV) analysis (Müller *et al.* 2018) showed strong evidence for aberrant CNVs for this additional cluster (Fig. 5c), supporting the presence of malignant cells. Interestingly, by performing gene set scoring using VAM (Frost 2020), we found that cells in this cluster were significantly enriched for Moffitt basal-like genes but were absent of Moffitt classical genes (Fig. 5d and e). This indicated that the cluster may consist of PDAC basal-like cells. We then used DECODER to calculate the basal-like and classical weights and the ratio between them (bcRatio) for each cell (Peng *et al.* 2019). DECODER weights are continuous and measure the extent of basal-like-ness and classical-ness. This newly identified cluster showed high basal-like weights and bcRatio, but low classical weights, confirming that this cluster was a basal-like cell cluster (Fig. 5f–h). Additionally, GATA6, which is thought to be a discriminating classical subtype marker (O’Kane *et al.* 2020), was found to be depleted in this cluster, further supporting that this is a basal-like cell cluster (Fig. 5i). This newly identified basal-like cell cluster was composed of only 148 cells, comprising just 0.59% of the entire dataset. Therefore, this demonstrated that SCISSORS-identified subclusters of cells that showed strong evidence of being the basal-like tumor cells, which were rare in the dataset and of high biological interest but went undetected in the original study.

To investigate the potential utility of SCISSORS scRNA-seq-derived genes for analyzing bulk RNA-seq samples, we identified the top 10 basal-like and classical genes ranked by the fold-change of averaged gene expression. There were three basal-like and three classical genes that were overlapped between the Moffitt genes and SCISSORS genes. Applying the SCISSORS tumor genes to TCGA pancreatic adenocarcinoma (PAAD) dataset, we found that the patients were clustered into two subtypes (Fig. 5j) (Raphael *et al.* 2017). Compared with published calls by the Moffitt schema (Rashid *et al.* 2020), SCISSORS-based subtype calls showed 139 (33 basal-like and 106 classical) consistent calls out of 150 samples (Fig. 5j). Similar to TCGA PAAD, patients with basal-like tumors subtyped by SCISSORS genes showed significantly shorter survival (*P* = .025, hazard ratio = 1.723, and log-rank test) (Fig. 5k). Thus, marker genes derived from SCISSORS *de novo* can be used to derive consistent patient subtypes in bulk RNA-seq samples.

### 3.6 Pinpointing overlooked apCAF cells in a PDAC dataset

**Figure 6.**
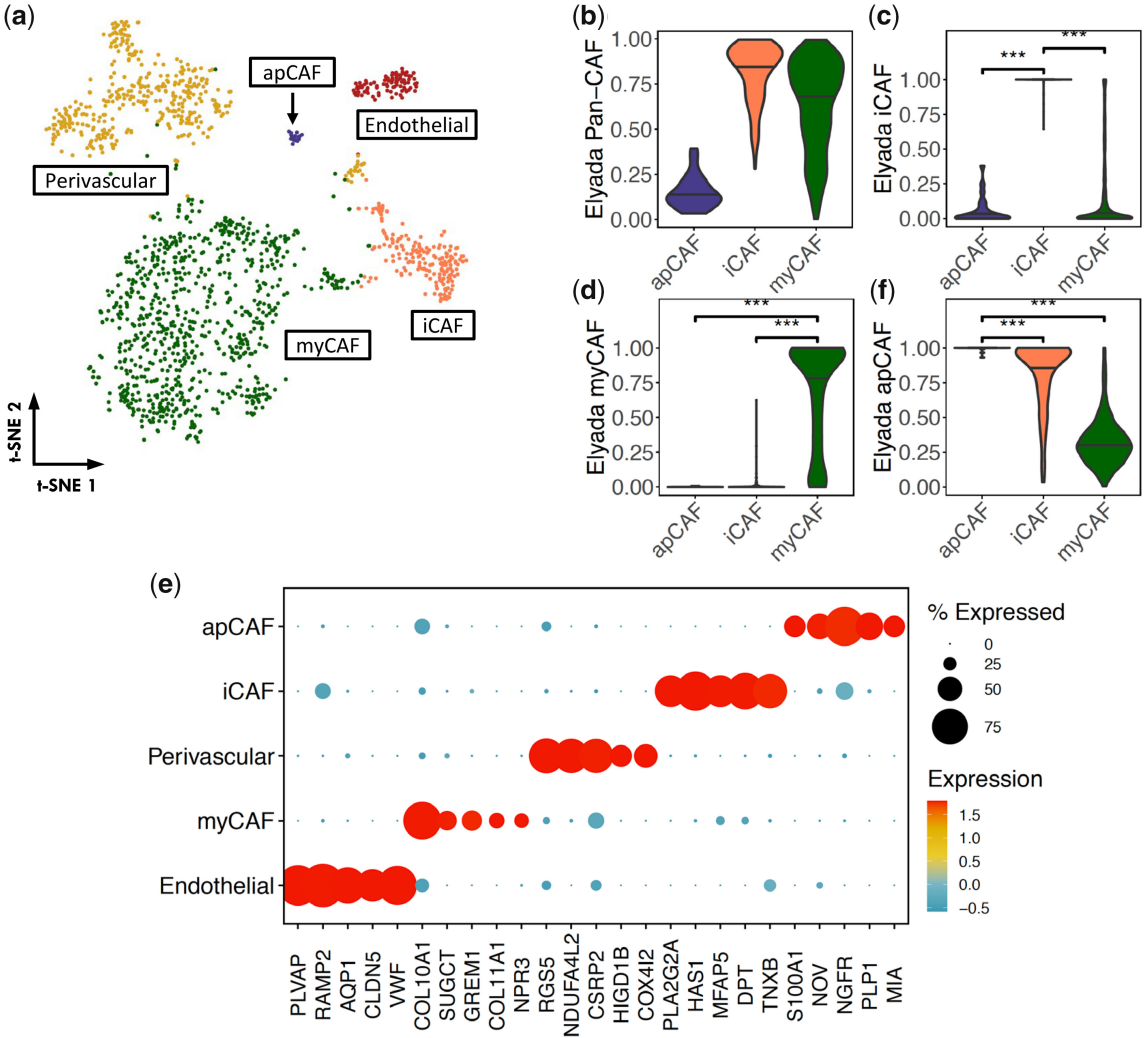
Pinpointing overlooked apCAF cells in the PDAC dataset. (a) Reclustering of the fibroblast cell clusters revealed five subclusters. (b–d and f) Gene enrichment analysis for each cell in the three fibroblast subclusters, using VAM scores based on Elyada pan-CAF, iCAF, myCAF, and apCAF genes. An apCAF cell subcluster was identified showing apCAF gene enrichment. (e) Top 5 marker genes identified by SCISSORS, ranked by mean log2 fold-change. ***P < .05, Wilcoxon rank-sum test

In PDAC, cancer associated fibroblasts (CAFs) have been shown to be heterogenous (Öhlund *et al.* 2017, Elyada *et al.* 2019). In the same PDAC dataset, we applied SCISSORS on Cluster 8, denoted as activated stellate/mesenchymal stem cells by SingleR, to identify CAF subtypes within the larger fibroblast population (Supplementary Fig. S2a). Unlike the original study, the endothelial and perivascular cells were clustered within our initial activated stellate/mesenchymal stem cell group (Fig. 6a and Supplementary Fig. S2a) likely due to the conservative parameters, we used in the initial clustering step. In addition to the endothelial and perivascular cells, we derived three additional subclusters, all of which showed enriched pan-CAF gene expression (Fig. 6b). Among them, two of the subclusters exhibited strong enrichment of inflammatory CAF (iCAF) and myofibroblastic CAF (myCAF) genes, respectively (Fig. 6c and d), which aligned with the discovery of iCAF and myCAF populations in the original study (Elyada *et al.* 2019). Interestingly, marker genes for the novel cluster identified by SCISSORS exhibited a large overlap with the antigen-presenting CAF (apCAF) subtype described previously (Elyada *et al.* 2019) (Fig. 6e). After running an enrichment analysis of the originally defined apCAF marker genes using VAM (Frost 2020), we saw that cells in this cluster showed significant enrichment (Fig. 6f), confirming their apCAF identity. In the original study by Elyada *et al.*, the apCAF population was only explicitly defined in scRNA-seq samples from KPC mouse models. Although the existence of the apCAF subtype was validated through mass cytometry staining of human PDAC sections, they were not identified in the original human PDAC scRNA-seq data analysis. Re-analysis using SCISSORS of the same human PDAC scRNA-seq data was able to identify this rare apCAF population; the apCAF cluster contained 23 cells and made up only 0.092% of the entire dataset. Thus, we again demonstrated that SCISSORS enabled the identification of extremely low abundance cells, such as apCAFs, which were not identified in the original study.

## 4 Discussion

Most clustering methods, perform a single clustering step on the entire dataset, seeking to estimate parameters that are optimal for the detection of all cell types and subtypes present in the data simultaneously. In the case of Louvain modularity optimization, which SCISSORS uses, this means determining a single set of values of the number of nearest neighbors *k* and resolution *r* that both keep large and homogeneous clusters intact and separate out rare cells that might have only faint differences in gene expression from related cell types. Decreasing *k* or increasing *r* generally results in the identification of rare cells at the expense of the overclustering of truly homogeneous groups into spurious or biologically uninteresting clusters. The inverse also occurs; increasing *k* or decreasing *r* usually leads to the preservation of homogeneous clusters along with the grouping of rare cell types into closely neighboring clusters. The application of SCISSORS eliminates the need to determine universally optimal values of *k* and *r* by providing an objective and systematic measure through the silhouette score and allowing the user the flexibility to determine if each cluster is heterogenous enough to necessitate reclustering. Furthermore, unlike other iterative-based methods e.g. MultiK (Liu *et al.* 2021), the iterative approach in SCISSORS is relatively time-efficient (median = 4.5 min on datasets composed of 10 000 cells) because it evaluates parameter sets in parallel and is only specifically applied on selected broad clusters. The method of CellSIUS involved a similar approach as SCISSORS, where a first round of clustering is performed followed by a second round of rare cell identification (Wegmann *et al.* 2019). However, SCISSORS has the flexibility of integrating more than two rounds of clustering if needed; in addition, it showed greater performance in identifying rare cells in the simulation test, as well as in the application to the PBMC3K dataset.

SCISSORS takes advantage of the silhouette score to systematically measure the cluster heterogeneity, which mimics the biological intra-cluster heterogeneity. When a ground truth biological state is not available, there are multiple metrics to measure the cluster quality. For example, within-cluster sum of squares and Davies–Bouldin index measure the variance within and between clusters; and Calinski–Harabasz index focus on the similarity between clusters (Hubert and Arabie 1985, Pedregosa *et al.* 2011). In contrast, the silhouette score provides a more comprehensive measurement that combines all of these aspects into a single, per-observation score (Rousseeuw 1987). A higher silhouette coefficient indicates that clusters are well-separated from each other and the data points within each cluster are tightly grouped together, and *vice versa*. Furthermore, compared to entropy-based metrics, silhouette score is insensitive to cluster size and shape, which better fit the true biological situation, where clusters are of variable sizes and shapes (Hubert and Arabie 1985, Pedregosa *et al.* 2011, Liu *et al.* 2020). Therefore, the metric of silhouette score was purposely chosen by SCISSORS among other clustering evaluation metrics. In addition, we purposely chose to use the relative cosine distance in our implementation because of its suggested better performance on higher dimensional data than Euclidean distance (Aggarwal *et al.* 2001, Banerjee *et al.* 2005).

SCISSORS generates a more biologically relevant clustering by re-selecting HVGs within each broad cluster. Rare cells appear infrequently among an ocean of other cell types, and their marker genes may be present as faint signals that might be excluded at the very beginning of an analysis due to the lack of expression in the majority of the cells and thus a low overall variance in expression. SCISSORS reconsiders all detected genes when performing reclustering in order to include genes that may be high variance within a broad cluster but are not considered as HVGs in the initial round of clustering.

In addition to the efficient identification of rare cells, SCISSORS includes a carefully optimized method for the identification of marker genes for cell clusters or subclusters. In the first comparison step, by comparing rare cells only to their closely related cells in the same broad cluster, distinguishing marker genes between them can be identified, which are often of higher biological interest. In the second filtering step, SCISSORS removes highly expressed genes in the clusters that are not of interest, which excludes potentially co-expressed genes to make the marker genes as specific as possible. With cell type-specific exemplar genes derived by SCISSORS, future validation studies can be performed, including the identification of cell types on other platforms (e.g. flow cytometry), and estimation of cell proportions in bulk samples.

Intuitively, rare cell identification may benefit from starting with a larger dataset. In this sense, large-scale data integration may increase the number of rare cells to be identified more effectively. With this kind of large-scale data, SCISSORS may display an advantage again by only reclustering on selected broad cell types to avoid overclustering on other clusters. SCISSORS supports the usage of integrated datasets as input, with re-integration being performed across samples within each reclustering candidate. The PCA subspace is recalculated in this setting. However, batch effects on these re-integrated data are a potential issue, which may hinder the effective clustering of the same rare cells across different studies. The batch correction issue is not specific to SCISSORS analysis and is being actively studied in the field of scRNA-seq analysis.

SCISSORS is applicable to a wide range of data scales theoretically. In this study, the smaller real dataset is the PBMC3K dataset with 2700 cells, and the larger real dataset is the Elyada dataset with around 25 000 cells. Our simulation datasets range in size from 1000 to 10 000 cells. Theoretically, SCISSORS has no restriction on input dataset size. Nevertheless, we recommend using SCISSORS on datasets that are of similar scales as these tested datasets.

## Supplementary Material

btad449_Supplementary_Data

## Data Availability

The PDAC scRNA-seq data from the study by Elyada et al. (2019) was obtained on NCBI dbGaP (accession number phs001840.v1.p1). The 10X Genomics PBMC3K dataset was obtained using the “SeuratData” R package (Satija et al. 2019). The Cancer Genome Atlas (TCGA)-normalized RNA-seq gene expression data were obtained from the Broad Institute FIREHOSE portal (http://gdac.broadinstitute.org).
