# Supplementary material for: Sub-Cluster Identification through Semi-Supervised Optimization of Rare-Cell Silhouettes (SCISSORS) in single-cell RNA-sequencing
Source: Bioinformatics. 2023 Jul 27;39(8):btad449. doi: 10.1093/bioinformatics/btad449 (PMC10412410; doi:10.1093/bioinformatics/btad449)
Supplement: btad449_Supplementary_Data [file btad449_supplementary_data.zip › Supplementary_Methods.docx]

## Supplementary Methods

## Initial clustering

Data preprocessing is performed using Seurat. Gene expression is normalized using regularized negative binomial regression as implemented in SCTransform, which also provides variance estimates for each gene (Hafemeister and Satija 2019). If desired, traditional log-normalization can be used as well. The top 4,000 highly variable genes (HVGs) are used to reduce the dimensionality of the gene-cell matrix through PCA (Hotelling 1933), after which the first 20 principal components are used as an initialization for a two-dimensional Fast Fourier Transform-accelerated t-SNE embedding (Maaten and Hinton 2008; Linderman *et al.* 2019; Poličar, Stražar and Zupan 2019, 2023). The cells are clustered in PCA space using Louvain modularity optimization after being embedded in a shared nearest-neighbors graph. For the PBMC3K dataset, *k*=52 (the square-root of the number of cells in the dataset) and *r*=0.4 are used. For the PDAC dataset, *k*=155 (the square-root of the number of cells in the dataset) and *r*=0.4 were used.

This step can be automatically performed by SCISSORS without the need to set any predefined parameters. This is because this first round of clustering can be far from perfect, with the aim being roughly separating the broad cell types. In our analysis, there is high tolerance of parameter choosing in this step to successfully separate the datasets into clusters of broad cell types, e.g., tumor cell, fibroblast cell, endothelial cell, T-cell etc. Cell clusters can be annotated by packages like SingleR for cell type assignment, or the Variance-adjusted Mahalanobis (VAM) method for gene set scoring in order to better associate biological information (Aran *et al.* 2019; Frost 2020).

## Reclustering

After cells are clustered and broadly defined in the initial round of clustering, one or more rounds of reclustering are used to identify subclusters within candidate broad cluster(s). The candidate broad cluster(s) for reclustering can be determined either automatically or in a supervised manner. Firstly, when biological information is limited, users may choose to allow the automatic determination of reclustering candidates by SCISSORS, which are chosen based on the derived silhouette scores for determining the heterogenous broad clusters. Secondly, when users have biological information on the heterogeneity of certain cells or have cell types that are of high biological interest, e.g., tumor cells, they may specifically apply reclustering on these cell groups using SCISSORS. Lastly, users may determine the reclustering candidates using a combination of biological knowledge and the silhouette score distributions. To determine the number of rounds needed, the following two criteria can be considered. First is if the final clusters are biologically desired; second is if the final clusters are too homogenous to be reclustered, which can be estimated by the silhouette scores. In the selected broad cluster(s), all the genes are re-normalized to select a new list of 4,000 HVGs. Dimension reduction is performed similarly to the pre-processing in the initial clustering step.


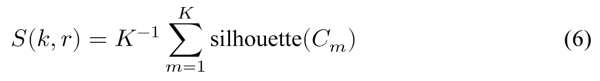
Then, SCISSORS iterates over a user-defined set of possible values for the *k* nearest-neighbors and resolution *r* parameters, which are used to embed the cells into a shared nearest-neighbors (SNN) graph and sort them into clusters. The default setting of SCISSORS is to compare three *k* and four *r* values, leading to a total of twelve clustering results. Each result is evaluated by computing the silhouette coefficient *S* of the dataset, which is derived by averaging the silhouette coefficients of each sub-cluster:

(6)

where *K* is the estimated number of clusters found using each parameter set. After recording the silhouette coefficient *S* for each set of clustering parameters, the maximum score is compared to a user-defined minimum threshold for reclustering (0.25 by default). If the best score exceeds that threshold, the accompanying reclustering is returned, else the original broad clustering is maintained. The threshold serves as a decision boundary for whether reclustering provides a noticeable improvement in fit for the candidate cluster; this parameter can be tuned, but in practice, values within [0.1, 0.3] are reasonable. If the default reclustering threshold is left unchanged, running SCISSORS to estimate the optimal values of *k* and *r* can be considered totally unsupervised. Possible optimal parameters are pre-defined empirically based on our past experiences. If the reclustering did not achieve expected performance, SCISSORS also allows users to redefine the set of potential values for *k* and *r*.

## Simulation analysis

A human lung cancer scRNAseq dataset (Zilionis *et al.* 2019), as well as a healthy human pancreas scRNAseq dataset (Baron *et al.* 2016) were used as references to generate simulation datasets using the R package ‘Scaffold’(Bacher *et al.* 2022). Scaffold accurately simulates sparse, UMI-based scRNA-seq counts; by fine-tuning the parameters, it was able to generate more complex datasets with challenging rare cell cluster topologies. Twenty-four datasets in total, with 12 for each reference, were generated using combinations of different number of clusters, cells, frequencies of differentially expressed (DE) genes and fold-changes of DE genes. To facilitate version control and manage stochasticity in the simulations, the ‘targets’ R package was used to implement a reproducible simulation pipeline (Landau 2021).

Seven comparable or basic clustering algorithms were involved in comparison with SCISSORS using simulated data, including Seurat (Louvain clustering) (Blondel *et al.* 2008; Hao *et al.* 2021), GiniClust3 (Dong and Yuan 2020), CellSIUS (Wegmann, et al., 2019), k-means clustering (Hartigan and Wong 1979), hierarchical clustering with Ward’s criterion (Ward 1963), DBSCAN (Ester *et al.* 1996), and Leiden clustering (Traag, Waltman and van Eck 2019). Multiple runs of each method were used on each dataset, iterating over the parameter space of each method to estimate performance over common and reasonable parameter values (Supplementary Table S2). In addition, during each run multiple metrics were generated and recorded for a systematic comparison, including cluster’s agreement with the ground-truth labels via the adjusted Rand index (ARI) (Rand 1971), normalized mutual information (NMI) (Vinh, Epps and Bailey 2009), and silhouette score (Rousseeuw 1987).

## Application of default Seurat, SAFE-clustering and GiniClust3 to PBMC3K

The default Seurat parameters were used to process the PBMC3K dataset as described in the Seurat (v3) PBMC3K tutorial (https://satijalab.org/seurat/articles/pbmc3k_tutorial.html). Briefly, two thousand HVGs were selected using the variance-stabilizing transformation method and then used as input to PCA. The cells were then clustered in PCA space with via the Louvain algorithm on 10 principal components using *k* = 20 nearest-neighbors and resolution *r* = 0.5.

In SAFE-clustering, SC3, Seurat, and t-SNE + k-means were used to create an ensemble clustering, with the filtered counts matrix as input. The optional CIDR method was not used as doing so is no longer considered best practices as per the authors’ recommendation (personal correspondence). For the SC3 clustering, the default parameters were used. The optimal value of *k* was chosen automatically, and the cells were clustered using k-means on the eigenvectors of the distance matrices (Kiselev *et al.* 2017). For the Seurat method, cells were clustered in 15-dimensional PCA space using *k* = 20 nearest-neighbors and *r* = 0.7. For the t-SNE + k-means clustering, the per-cell counts were transformed to counts per million mapped reads (CPM) and embedded in 3-dimensional t-SNE space with the perplexity set to 30 (Maaten and Hinton 2008). The optimal value of *k* was determined using adaptive density peak detection (Wang and Xu 2017). The final optimal value of *k* chosen during the ensemble clustering was estimated to be equal to 9.

GiniClust3 was used to cluster the cells using the normalized and filtered counts matrix as input, as described (Dong and Yuan 2020). *k* = 7 nearest neighbors were used when clustering the cells based on their Gini indexes. Default parameters were used when clustering the cells based on their Fano factors. The final consensus clustering was also generated using default parameters.

The identified clusters from different methods were annotated using canonical marker genes from the literature. A silhouette score for each cell in each cluster derived from each method was computed for comparison.

## Bulk RNA-seq analysis

Using the SCISSORS function for marker gene identification, PDAC basal-like and classical genes were identified *de novo* from the Elyada PDAC dataset. Specifically, the classical 1 and classical 2 clusters are combined as a classical cluster for comparison with the basal-like cluster (Supplementary Table S2). The identified marker genes are ranked by the fold-change between the averaged gene expression of the two groups for comparison. The top 10 ranked genes for the basal-like and classical cells respectively are then retained for further analysis.

The Cancer Genome Atlas (TCGA) pancreatic adenocarcinoma (PAAD) was used to call tumor subtypes (Raphael *et al.* 2017). Data was processed for the Moffitt schema and PurIST calls as described before (Peng *et al.* 2019; Rashid *et al.* 2020). Unsupervised consensus clustering (k-means) was applied on the distance matrix (Pearson) of the log2-transformed data using the top 10 SCISSORS-derived tumor genes using the ‘ConsensusClusterPlus’ R package v1.56.0 (Wilkerson and Hayes 2010). Overall survival was analyzed by log-rank test (Mantel 1966).

References

Aran D, Looney AP, Liu L *et al.* Reference-based analysis of lung single-cell sequencing reveals a transitional profibrotic macrophage. *Nat Immunol* 2019;**20**:163–72.

Bacher R, Chu LF, Argus C *et al.* Enhancing biological signals and detection rates in single-cell RNA-seq experiments with cDNA library equalization. *Nucleic Acids Res* 2022;**50**:e12.

Baron M, Veres A, Wolock SL *et al.* A Single-Cell Transcriptomic Map of the Human and Mouse Pancreas Reveals Inter- and Intra-cell Population Structure. *Cell Syst* 2016;**3**:346-360 e4.

Blondel VD, Guillaume J-L, Lambiotte R *et al.* Fast unfolding of communities in large networks. *J Stat Mech* 2008;**2008**:P10008.

Dong R, Yuan GC. GiniClust3: a fast and memory-efficient tool for rare cell type identification. *BMC Bioinformatics* 2020;**21**:158.

Ester M, Kriegel H-P, Sander J *et al.* A density-based algorithm for discovering clusters in large spatial databases with noise. *Proceedings of the 2nd International Conference on Knowledge Discovery and Data Mining* 1996;**96**:226–31.

Frost HR. Variance-adjusted Mahalanobis (VAM): a fast and accurate method for cell-specific gene set scoring. *Nucleic Acids Res* 2020;**48**:e94.

Hafemeister C, Satija R. Normalization and variance stabilization of single-cell RNA-seq data using regularized negative binomial regression. *Genome Biol* 2019;**20**:296.

Hao Y, Hao S, Andersen-Nissen E *et al.* Integrated analysis of multimodal single-cell data. *Cell* 2021;**184**:3573-3587 e29.

Hartigan JA, Wong MA. Algorithm AS 136: A K-Means Clustering Algorithm. *Journal of the Royal Statistical Society* 1979;**28**:100–8.

Hotelling H. Analysis of a complex of statistical variables into principal components. *Journal of Educational Psychology* 1933;**24**:417–41.

Kiselev VY, Kirschner K, Schaub MT *et al.* SC3: consensus clustering of single-cell RNA-seq data. *Nat Methods* 2017;**14**:483–6.

Landau WM. The targets R package: a dynamic Make-like function-oriented pipeline toolkit for reproducibility and high-performance computing. *Journal of Open Source Software* 2021;**6**:2959.

Linderman GC, Rachh M, Hoskins JG *et al.* Fast interpolation-based t-SNE for improved visualization of single-cell RNA-seq data. *Nat Methods* 2019;**16**:243–5.

Maaten L van der, Hinton GE. Visualizing Data using t-SNE. *Journal of Machine Learning Research* 2008;**9**:2579–605.

Mantel N. Evaluation of survival data and two new rank order statistics arising in its consideration. *Cancer Chemother Rep* 1966;**50**:163–70.

Peng XL, Moffitt RA, Torphy RJ *et al.* De novo compartment deconvolution and weight estimation of tumor samples using DECODER. *Nat Commun* 2019;**10**:4729.

Poličar PG, Stražar M, Zupan B. *OpenTSNE: A Modular Python Library for t-SNE Dimensionality Reduction and Embedding*. Bioinformatics, 2019.

Poličar PG, Stražar M, Zupan B. Embedding to reference t-SNE space addresses batch effects in single-cell classification. *Mach Learn* 2023;**112**:721–40.

Rand WM. Objective criteria for the evaluation of clustering methods. *Journal of the American Statistical Association* 1971;**66**:846–50.

Raphael BJ, Hruban RH, Aguirre AJ *et al.* Integrated Genomic Characterization of Pancreatic Ductal Adenocarcinoma. *Cancer Cell* 2017;**32**:185-203.e13.

Rashid NU, Peng XL, Jin C *et al.* Purity Independent Subtyping of Tumors (PurIST), A Clinically Robust, Single-sample Classifier for Tumor Subtyping in Pancreatic Cancer. *Clin Cancer Res* 2020;**26**:82–92.

Rousseeuw PJ. Silhouettes: A graphical aid to the interpretation and validation of cluster analysis. *Journal of Computational and Applied Mathematics* 1987;**20**:53–65.

Traag VA, Waltman L, van Eck NJ. From Louvain to Leiden: guaranteeing well-connected communities. *Sci Rep* 2019;**9**:5233.

Vinh NX, Epps J, Bailey J. Information theoretic measures for clusterings comparison: is a correction for chance necessary? *Proceedings of the 26th Annual International Conference on Machine Learning* 2009:1073–80.

Wang X-F, Xu Y. Fast clustering using adaptive density peak detection. *Stat Methods Med Res* 2017;**26**:2800–11.

Ward JH. Hierarchical Grouping to Optimize an Objective Function. *Journal of the American Statistical Association* 1963;**58**:236–44.

Wilkerson MD, Hayes DN. ConsensusClusterPlus: a class discovery tool with confidence assessments and item tracking. *Bioinformatics* 2010;**26**:1572–3.

Zilionis R, Engblom C, Pfirschke C *et al.* Single-Cell Transcriptomics of Human and Mouse Lung Cancers Reveals Conserved Myeloid Populations across Individuals and Species. *Immunity* 2019;**50**:1317-1334 e10.
