# Supplementary material for: Sub-Cluster Identification through Semi-Supervised Optimization of Rare-Cell Silhouettes (SCISSORS) in single-cell RNA-sequencing
Source: Bioinformatics. 2023 Jul 27;39(8):btad449. doi: 10.1093/bioinformatics/btad449 (PMC10412410; doi:10.1093/bioinformatics/btad449)
Supplement: btad449_Supplementary_Data [file btad449_supplementary_data.zip › Supplementary_Figures.pdf]

Supplementary Fig. S1

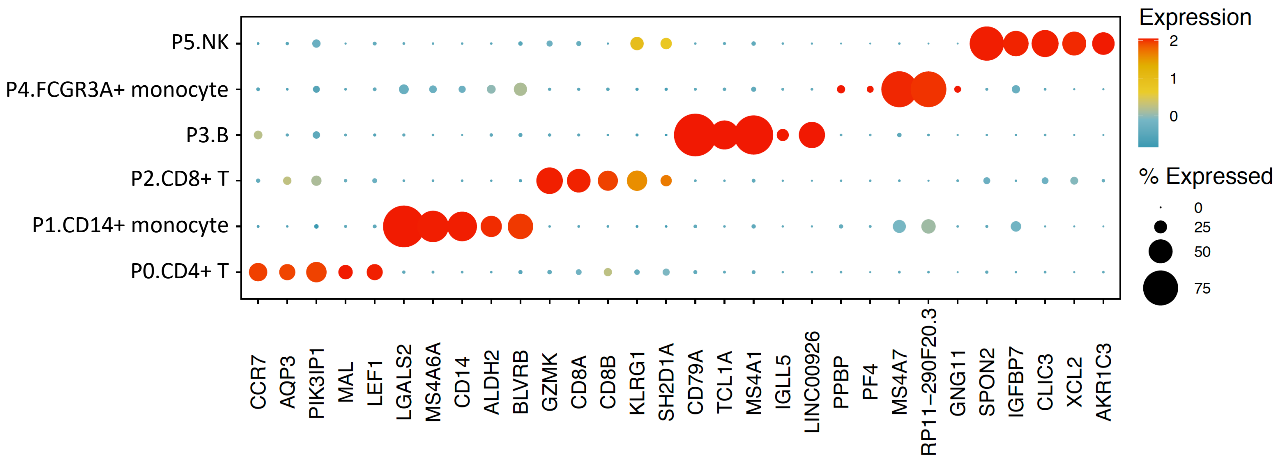

Supplementary Fig. S1. Top 5 marker genes ranked by fold-change for the major clusters identified by SCISSORS in the PBMC3K dataset.

Supplementary Fig. S2

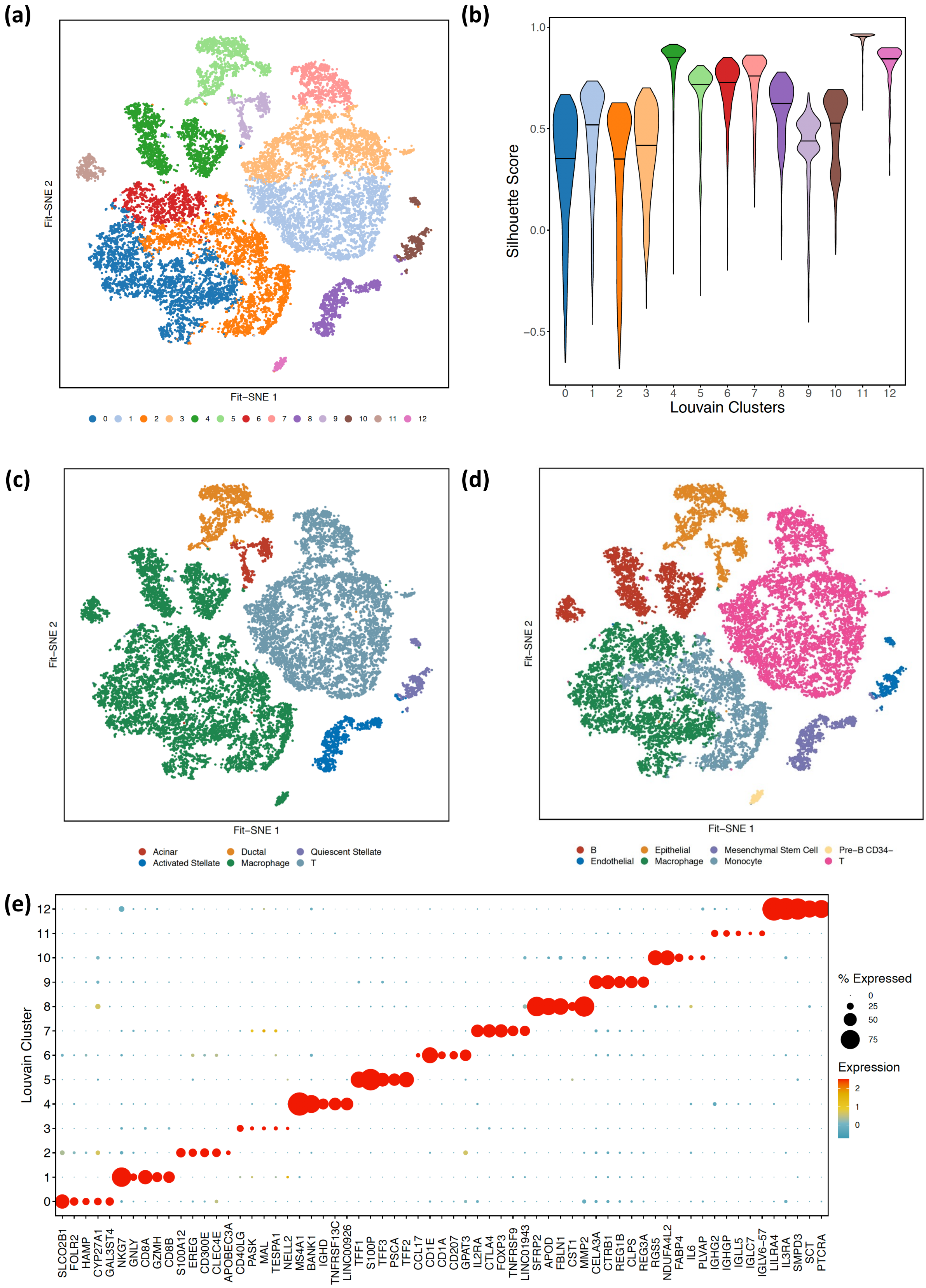

Supplementary Fig. S2. Application of SCISSORS to the Elyada PDAC dataset. (a) Initial conservative clustering. Twelve major clusters were identified. **(b)** Violin plot for silhouette scores in each major clusters. **(c) & (d)** SingleR annotations on the major clusters using the single-cell PDAC dataset and bulk human cells as references. **(e)** Top 5 marker genes for the major clusters identified by SCISSORS, ranked by fold-change.
